# Physical Spaces in Higher Education as Scenarios of Learning Innovation: Compositional and Formative Synergies among Architecture, Music, and Fashion

**DOI:** 10.3390/ejihpe11040086

**Published:** 2021-10-01

**Authors:** Pablo Campos, Laura Luceño, Carlos Aguirre

**Affiliations:** 1 Escuela Politécnica Superior, Universidad CEU San Pablo, 28003 Madrid, Spain; utoplan@telefonica.net; 2 Centro Superior de Diseño de Moda de Madrid (CSDMM), Universidad Politécnica de Madrid, 28040 Madrid, Spain; laura.luceno@telefonica.net or; 3 Escuela Internacional de Doctorado CEINDO, Universidad CEU San Pablo, 28003 Madrid, Spain; car.aguirre.ce@ceindo.ceu.es

**Keywords:** higher education, architecture, music, fashion, learning innovation, composition, areas of knowledge

## Abstract

Learning innovation is a positive approach on the contemporary higher education international stage. This article stresses the need to devise physical spaces that are also innovative. For that purpose, using a qualitative methodology, we investigated recent trends based on the synergies between certain creative disciplines: architecture, music, and fashion. The comparison was based on compositional features and formative dimension. Using a qualitative methodological comprehensive approach, a set of case studies was analysed. The findings show the usefulness of activating these synergies as effective strategies when enriching educational processes in different ways. Six cases of excellence wherein university physical spaces reached levels of innovation were studied, representing relevant transfers among the three disciplines. The text presents examples that show the educational consequences in the establishment of those synergies, in terms of both composition (music–architecture) and the activation of heritage sites in the city as venues of learning innovation (fashion–architecture). The basic conclusions were based on the fact that the increase in teaching and spatial creativity that emanates from said synergies among the three disciplines can be potentially extrapolated to other areas of knowledge.

## 1. Introduction

### 1.1. Higher Education: Models through History

Throughout their historical evolution, higher education institutions have adopted different institutional, academic, and urban-architectural models [1]. Originally, the medieval model was characterized by its integration with the urban context, with the cloister (inherited from the monasteries and cathedrals) as the preferred spatial type. The modern model has been broken down into several trends. The English pattern was founded on the colleges of Oxford and Cambridge, which inherited cloister typology for their architectural structures. The French Napoleonic model sought an implantation within the city, consisting of a polycentric structure, in the Latin Quarter of Paris. This same pattern of urban integration characterized Humboldt University, inserted in the heart of Berlin since the beginning of the 19th century. The most important model was the North American campus. Having emerged as a legacy of the British colleges, in its transfer to the American continent, its evolution crystallized on the campus as an isolated model of the city, paradigmatic in the creation of vital communities within which various activities were developed: study, research, relationship, leisure, sport, and residence. In sum, the evolution of universities shows that there has always been a connection between academic philosophy and its urban and architectural body [2].

### 1.2. Learning Innovation and Architecture

Higher education needs to face continuous challenges in terms of innovation, which stimulates numerous processes of pedagogical transformation. This is highly relevant in the European Higher Education Area (EHEA), the academic setting on which this text is primarily focused. The EHEA still offers today a sound opportunity for innovation across multiple dimensions—teaching and learning modalities together with urban and architectural design. However, most of the administrative criteria have to date been academic, underestimating the interactions between educational activities and the physical spaces that host them. This would be the case for various documents, such as the Declaration of the Sorbonne [3] and the subsequent “Bologna Declaration”.

As has been widely demonstrated by pedagogists, faculty, and psychologists, well designed physical spaces acquire the power of fostering motivation in students for all educational activities [4]. This issue has been researched through decades, using a qualitative methodology that traditionally has been based on a comprehensive approach to the matter. Thus, there must be a close connection between innovation in higher education and architecture, as recent trends indicate [5]. Some point out that any environment can become a potential learning space if properly planned. This idea refers to a reality that must be differentiated from the traditional ambits. Currently, this aspect must be interiorized under a modern conceptual approach regarding the “transactions” of knowledge [6].

The physical context becomes critical in fostering modern pedagogical formats that go beyond the formal lecture [7]. One extremely relevant aspect should be human scale. The concept of “learning place” must replace “learning space”. The reason for this is that, in order to attain the full power of education, the human being has to activate some type of emotional connection with the physical environment. The aim is to achieve a kind of identification and sense of “belonging”—to build a “place”, so that the educational action itself enjoys physical, affective, and cultural roots. Conversely, it is the unfolding of learning itself that helps crystallize “place” as an agent that plays an active role in it.

### 1.3. Learning as a Spatial Experience: Interaction with the Environment

There is no formative innovation without spatial (urban and architectural) innovation. As a highly relevant human activity, education depends on interactions with environments both cultural and physical [8]. There are numerous scientific contributions that have defended the existing relationship between the aesthetic quality of the built environment, teaching innovation, and the subsequent motivation of the students [9,10]. In spite of that, it is quite evident that there is a normative void in relation to the constructed dimension of higher education, which is an unjustifiable lack, since architecture plays a crucial role in the activities of human formation (the influence of environment). In recent years, some scientific and practical works have tried to fill that void, emphasizing the necessary quality of the urban and architectural dimensions and, among them, the emerging concept of “educational campus” [11]. Understood as integral events, qualified learning processes need physical spaces in which to be properly performed. Those have traditionally involved four major scales: university-city, campus, building, and classroom. The present text was mainly centred on the former two, as they are exemplary of the two goals analysed: compositional and formative synergies among three creative disciplines (architecture, music, and fashion). 

## 2. Materials and Methods

### 2.1. Conceptual Materials: Synergies among Architecture, Music, and Fashion

As a consequence of the qualitative methodology carried out, the present text handled conceptual materials that are not susceptible to quantification. The article focused on the analysis of synergies among three creative disciplines: architecture, music, and fashion. Although diverse in their respective contents and specific professional orientations, they all share one essential goal: to accomplish integral education based on innovative learning modalities and associated physical spaces. There are historical inertias that demonstrate the links among these three disciplines. In the case of the architecture–music duo, cases can be remembered from the Renaissance, e.g., the theories of Alberti or the works of Guillaume Dufet (whose composition Nuper Rosarum Flores inaugurated the Florence Cathedral designed by Brunelleschi in 1436). The modern age witnessed much evidence for this synergy, such as the relation between Le Corbusier and Xenakis and, more recently, projects by Steven Holl inspired by Bartok’s *Music for Strings, Percussion, and Celesta*. The North American architect stated, “In music as well as in architecture, form, rhythm, proportion, and mathematics are of elementary importance” [12]. On the relationship between architecture and fashion, there is an abundant recent bibliography, which highlights the close compositional analogies between the two [13,14]. This led us to assume the urban projection of fashion and, therefore, its spatial component. These synergies among the three disciplines similarly affect the way they are taught to students, sharing a vocation for innovation inherent in their own creative nature [15,16]. There is also scientific background on the synergies between fashion and music [17]. Finally, it should be noted that the physical space is a common element among the three in regard to teaching innovation, as exemplified in compositional guidelines that inspire campus design and stimulate learning for students (architecture–music) and in criteria for the use of spaces and heritage elements of the city as recipients of educational innovation activities (fashion–architecture).

### 2.2. Conceptual Materials: Learning Innovation and Physical Space

At this point, we describe some basic pedagogic modalities that have emerged in contemporary higher education. A research project carried out in Spain using a qualitative methodology classified the most relevant ones, ordered from traditional formats to innovative patterns, explaining their educational features together with the spatial consequences:

1. traditional masterly lesson; 2. interactive masterly lesson; 3. lecture on panel; 4. polarized (seminar—global tutoring); 5. general idea sharing; 6. idea sharing by nuclei (seminar—partial tutoring); 7. polarized nuclei; 8. interactive session in multiple panels; 9. reflection in common “soft seat”; 10. working places; 11. scenographic simulation of real activity; 12. individual study; 13. individual tutoring; 14. distance education; 15. student presentations; 16. staging and learning supported in other arts; 17. on-site experience (guided visits); 18. individual contemplative learning; 19. mobile learning; 20. social learning; 21. work practices; and 22. community service [18].

These innovative learning modalities affect both pedagogical and physical aspects. Furthermore, such innovation demands new attitudes from the faculty (a renewed role that becomes clear in the case of architecture, music, and fashion). The teacher is no longer a mere transmitter of content, but a guide or advisor and facilitator in charge of guiding students and introducing innovative pedagogical modalities. It has become evident that human education processes acquire spatial shapes that correspond to those pedagogic innovations. Education has shapes [19]. 

### 2.3. Qualitative Methodology: Description and Justification. A Comprehensive Approach to Learning Synergies among Architecture, Music, and Fashion

Architecture, music, and fashion are creative disciplines. This recommends the use of a quantitative methodology in the research of their common features, as in the present text. Following a comprehensive approach, the article analysed the three disciplines studied, as well as six case studies, because they are not translatable into mathematical terms. Their respective teaching and learning modalities go together with a set of innovative spaces characterized by moving away from traditional formats: ideation workshops, performance venues, and outdoor spaces on campus and urban settings. Besides these common aspects, they share two quite relevant features: composition strategies and innovative education. In the first, comparison between musical and architectural features is used as a method, resulting in certain innovative stimuli to inspire the ideation of university campuses and enrichment of student’s design skills. In the second, certain formative trends in fashion (wherein music also intervenes) can generate potential transfers to architecture, using the city heritage reality as a stage.

In order to illustrate the compositional and formative similarities among the three disciplines, some concepts should be described. Equivalent to the idea of “elaboration”, composition is the foundation of all creative activities. Any artist embodies an idea after manipulating pre-existing materials, in accordance with organizational laws that guarantee that the result acquires coherence and unity. Historically, two global methods are recognizable in the elaboration of architecture (extrapolated to fashion and music): assembly and modelling [20]. The first talks about the combination of parts in the global work, while the second refers to the global treatment, a sort of “moulding”, so the final work is the outcome of a first global energetic impulse. Fashion and architecture are based on geometric elements, such as point, line, plane, and volume, although space is exclusive to the latter. Other common features are symmetry, balance, proportion, and order. Agglutinated around the composition process, the following features could be found, shared by music: rhythm, proportion, balance, limit, or ornament [21,22,23]. A complementary characteristic that should be present in any sound compositional method of a work is adaptation to the place, understood as the series of characteristics of the social and urban context of their time. As an example, when a complex addressed to host higher education lacks such sensitivity, the interference of foreign styles occurs; instead, the aim should be to fit in with local cultures [24]. What matters is achieving the goal of generating quality in the different works composed in the three disciplines. Finally, it must be remarked that quality in composition becomes a formative value in itself, as it transmits to whoever experiences a work the aesthetic value acquired in such a process of “elaboration”. 

Regarding education, it must be underlined that architecture, music, and fashion are taught making use of innovative pedagogical modalities that share common features, such as: project-based learning, learning supported in other arts, collaborative work, simulation of professional activity; or student presentations, among others. Because of their creative nature, the education corresponding to each of them also shares the use of historical context as a stimulus and a source of enrichment of knowledge for students. By way of illustration, innovative pedagogical practices should be remembered, such as the design studio shared by architecture and fashion [25].

### 2.4. Qualitative Methodology: The Case Study Applications

The present text was centred on a specific theoretical nucleus: the synergies between architecture, music, and fashion and the outcomes of said synergies that affect learning innovation (in aspects such as composition and formation). Consequently, we carried out an intensive and concentrated study on a small-scale scenario (described throughout the text). Once again, this justified the choice of the qualitative methodology, as it is interesting not to analyse a large population or global scenario (which would have corresponded to a quantitative methodology), but rather to study a few topics or cases in depth. The methodology used case studies through a comparative intention [26,27]. This system “explores a real-life, contemporary bounded system (a case) or multiple bounded systems (cases) over time, (…) and reports a case description and case themes” [28] (p. 97). This qualitative method was chosen for the present text because it is advantageous for developing theories and involvements [29]. This method is also used in other spheres, such as business, as well as in the analysis of fashion as an urban phenomenon in certain countries [30] and, without a doubt, in those approaches that are interested in innovation in teaching of music and architecture [31,32].

In the following sections, attention is paid to two types of synergies that are especially useful for promoting learning innovation in the three disciplines: in the case of the music–architecture relationship, the use of “expressive” and “morphological” trends to devise physical spaces on an urban scale (campus) characterized by their stamp of modernity; and in the fashion–architecture interaction, the potential of the city’s heritage as a training resource and a scenario for the social projection of student academic results.

## 3. Results

Outcomes of the qualitative methodology applied: Compositional and formative synergies between Architecture, music, and fashion and their impact on the design and use of physical spaces for learning innovation in higher education.

Architecture, music, and fashion share several compositional and formative features, as expressed earlier [33]. Next, we emphasize two facets of these synergies that provide considerable doses of innovation at the present time. We used a qualitative methodology to analyse the outcomes derived from the six case studies because of the method’s inductive profile and flexible characteristics, and because it genuinely seeks to understand situations (synergies among the three creative disciplines, in this case) rather than to establish a cause–effect relationship.

### 3.1. Musical Composition and Its Potential Influence As an Inspiration Resource for the Innovative Design of Spaces for Human Formation. A Qualitative Methodological Approach

Music and architecture share several artistic issues, including the second having been defended as a solidified version of the first (as noted by Schelling in the 19th century). When comparing them, it becomes evident that both artistic expressions have synergies, sharing various compositional features such as order, rhythm, pause, proportion, and module (which can also be identified in fashion). Musical compositional elements are resources used for creation. Schoenberg stated that “form means a piece that is organized, that is, that consists of elements that are integrated like those of a living organism...” [34] (p. 1). One common feature between architecture and music is the space–time dimension. Since music is played in present time, architecture is a three-dimensional reality (a characteristic also evident in fashion). However, as an artistic performance, the user of architecture needs time to be perceived; thus, architecture acquires a sort of fourth dimension. Somehow, a musical composition is a sort of architectural work in movement. The correct use of compositional elements is also part of the creative apprenticeship of composers in architecture, music, and fashion. Those elements create a language-like system of rules and notations that are divided into two trends: expressive and morphological. 

#### 3.1.1. Expressive Compositional Trend

Expression is the vivid manifestation of feelings. This can be observed in art through all kinds of means, each with its own language: mimesis, oral, written, musical, or visual. The different sensations aroused while listening to music, such as fear, happiness, or sadness, were analysed by Vieillard [35]. Expressivity is a central aesthetic issue that affects music and architecture. Its presence can also be identified in associated versions of creativity, such as fashion, which shares with architecture various types of spaces and teaching methodologies [36]. Recent experiences showed that the implementation of these creative educational strategies can involve synergies with cities (where architecture, music, and fashion can merge) [37].

Music manifests specific emotions of its authors using compositional elements that induce expressiveness in whoever experiences them—from rhythm or silence, as traditional elements of the narrative of the musical message, to *ritardando*, accelerating, or blurred tempo, as modern ones. Emphasis can be assigned to a certain element that is called to exercise a singular role. With this, what could be called an “ordered disorder” is generated, where elements are subordinated to certain points or moments that are more intense than the rest. From this perspective, there are many synergies with spatial expressiveness, as is explained later with some relevant cases of campuses.

#### 3.1.2. Morphological Compositional Trend

Musical morphology is described as the concrete graphic form that creates a system understood as a language. It has been claimed that the “crystallized” musical form is a kind of compositional model that defines a universal pattern. This approach clarifies the synergy between musical and architectural morphological genesis. The morphological elements in music are very diverse, and their combination makes it possible to create a language of its own that, like a living being, can grow, evolve and transform itself. The morphological elements are motif, phrase, pattern, tone, timbre or pitch, and their combinations with each other. All of these elements can be identified in the architectural design scenario after appropriate adaptation to its specific characteristics.

Some features of contemporary modalities of musical composition can be transferred to architecture as sources of inspiration, but they can also be used in Fashion. The combination of designs that present morphological or expressive characters is fully feasible. The ideation of certain garments can be based on Cartesianism in the making of the pattern, as well as a tribute to an element that prints expressive “character”. In architecture, the “character” of a project lies in its strength, originality, or (in a more poetic way) its capacity to “stir men’s blood”. Put differently, the central idea of the project—its essence or “soul”—should inscribe itself deeply upon the mind of its “consumers”. For this reason, the expressive “character” is present in the three disciplines.

- Relevant contemporary illustrations (campuses): outcomes of the qualitative methodology approach.

Once expressive and morphological trends were defined as compositional features shared by music and architecture, several cases were analysed to illustrate the common synergies. Those cases were chosen from among scenarios at higher education complexes because a campus represents a comprehensive physical entity, the essential goal of which is precisely the education of students. The multidimensional interest that a campus has justifies why a certain author understood it as a work of art [38]. Campuses are large-scale academic complexes with functional richness as receptacles for training activities. For this reason, creative strategies validate them as outstanding scenarios of formative processes for architecture, music, and fashion. The three examples chosen illustrate this argument. As an added argument, the three campuses are compared with three musical compositions, almost contemporaneous with them, in order to analyse works corresponding to similar periods of history.

#### 3.1.3. Illinois Institute of Technology Campus (Chicago, IL, USA)

**Scheme 1:**
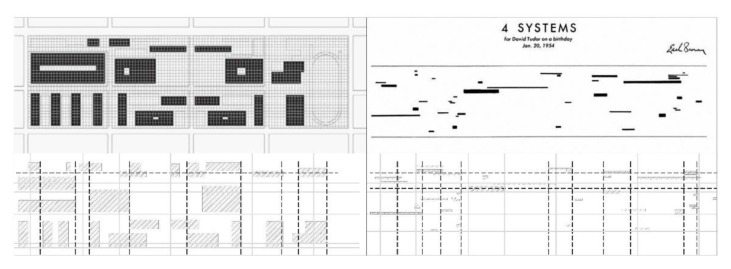
Compositional comparison: *4 Systems*–IIT Campus (source: authors).

The Illinois Institute of Technology campus, designed by Mies Van der Rohe in 1940, is quite an outstanding example of a university complex wherein synergies with music can be identified. The main pattern of the original plan is closely linked to Cartesianism. The campus, although representing International Style architectural principles, reflects a morphological composition that connects it with the Beaux-Arts trend in traditional North American campuses [39]. One of the essential features that links the layout of this technological campus with the morphological trends in music is the use of the orthogonal grid and its modulations, which generate both the minor units (buildings) and the global complex. The rhythmic use of the grid is also related to the technological profile of IIT. Another element that can be identified is fluency, a trait that emerges in contemporary technology campuses (where architecture and engineering are taught), a fluidity that is transferred to the design of the architectural pieces, as in the famous Crown Hall. A final look at IIT serves to underline the versatility in the space–time dimension of the campuses (to verify further analogies with musical compositions). Ordering guidelines coexist that are not excessively monolithic, but rather allow the competition of other modalities of formal ideation. Because of its rational identity, in the composition of the IIT campus, two trends merged: axiality, as a legacy of classical Architecture, and the freedom inherent in the Modern Movement. As shown in the attached Scheme 1, it is feasible to set up a compositional comparison between the IIT Campus and the musical work *4 Systems*, created in 1954 by Earle Brown. As its main feature, *4 Systems* is performed with shapes, without breaks, with only interlacing and interweaving among elements. 

#### 3.1.4. Aalto University Campus (Espoo, Finland)

**Scheme 2:**
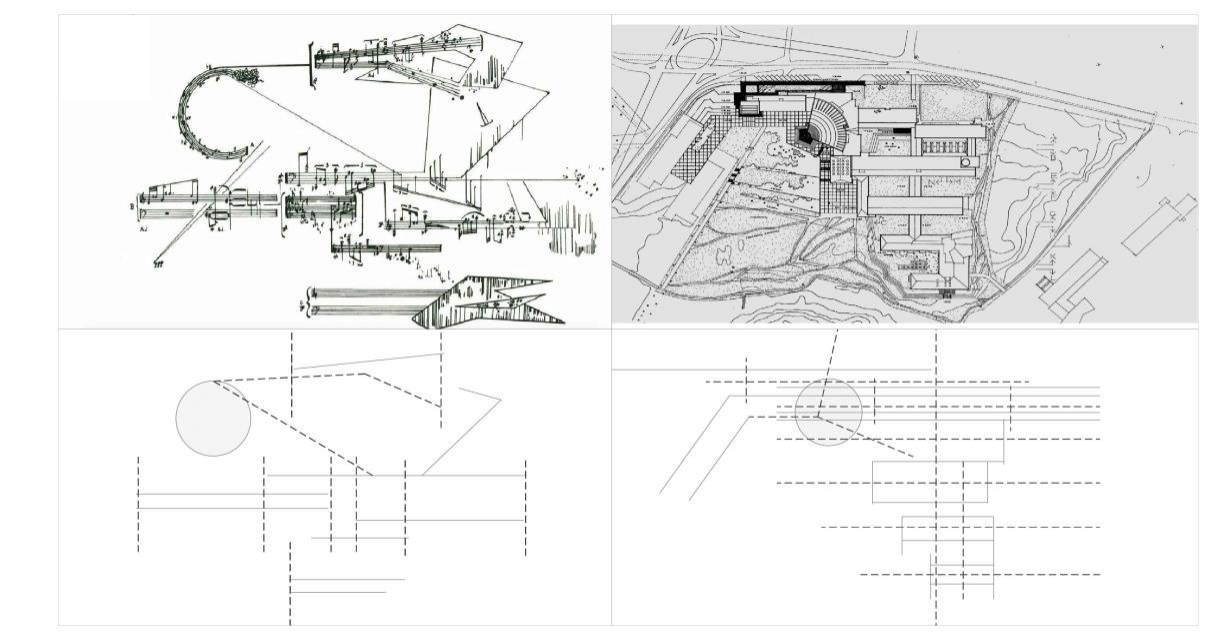
Compositional comparison: *Notations*–Aalto Campus (Source: authors).

The Aalto University campus (formerly TKK) represents a sound example of the expressive trend of translation from music to architecture. Its genesis dates to 1948, when the decision was made to move the pre-existing university to Espoo. The variety of forms of this university complex coexists with the predominance of a cardinal nucleus (the pieces designed by Aalto) and the weight of the void (free space) as a compositional element. Under a functional prism, the campus combines science and art together with technology, engineering, and business under the organicist principles of the Finnish master. The expressive vitality of this project has its origin in the author himself, who adopted a clear attitude of flexibility regarding the functional programme [40]. As a metaphor for expressive trends in music, the central area pays homage to “ordered disorder” by acting as a crossroads of two influences: the American prototype (with its emphasis on landscaping) and the residential areas (the planning Scheme 2 of which bears similarities to Aalto’s design for the Sunila complex). Back to the expressive synergies between music and architecture, it must be underlined that the natural component is also present in the morphological inspiration of various built pieces of the Finnish case. Spatial harmony reaches a specific point of great expressiveness in the iconic volume of the Aula Magna, the Polytechnic, the Library, and the adjacent free space. The musical work *Notations*, created by John Cage in 1968, shares compositional features with the Aalto campus, as expressed in the attached graphic. The melody is an example of typesetting composition using chance-derived mixtures of typefaces and sizes. 

#### 3.1.5. Free University of Berlin Campus (Berlin, Germany)

**Scheme 3:**
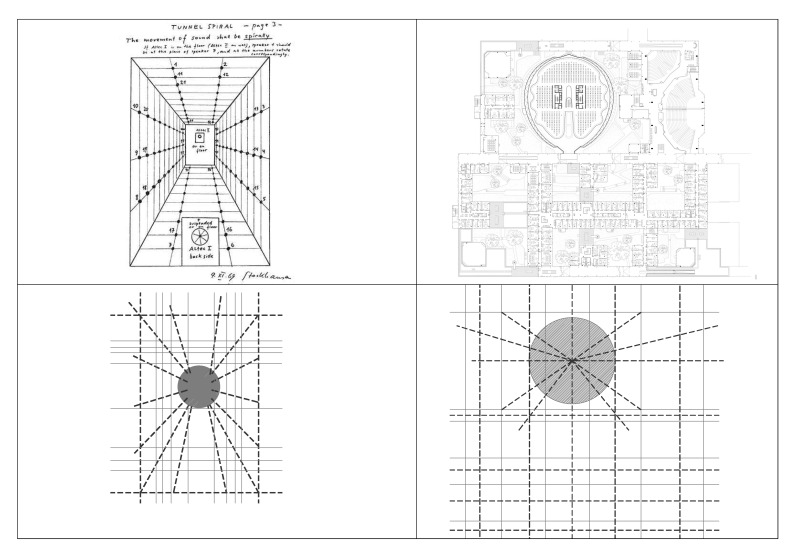
Compositional comparison: *Tunnel Spiral–*FUB Campus (Source: authors).

The Free University of Berlin (FUB) is a modern institution in Germany. Its built complex encompasses the two compositional trends of innovation in music: expressive and morphological. Founded in 1948, it represents a transcendent planning solution, a true paradigm of a spatial grid wherein the traces of a Cartesian pattern can be identified. The origins of this campus date back to 1963, when Candilis, Josic, and Woods (members of “Team X”) composed a highly innovative spatial structure. After decades of evolution, in 2005, the insertion of the new Library, designed by Norman Foster, represented a milestone in the campus transformation, establishing a contrasted dialogue between the reticular model and the library as a singular oval element. The emergence of the new architectural object introduced a compositional dynamic fully similar to the expressiveness of music [41]. To this can be added certain specific connections with musical works, such as the pieces *La Passion Selon Sade* (Bussotti, 1966) or *Tunnel Spiral* (Stockhausen, 1968), wherein geometric forms become graphic musical signs. The ideation of these pieces can be linked to the structural module of the FUB, as well as the modern radial axes in the library plan. This type of composition draws a systemic set of notations related to principles of hierarchy, order, symmetries, and tempos. In the FUB campus, a gradual implementation of educational modern modalities has been introduced. Social learning floods the entire system of walks, interior streets, and meeting spaces. The architectural uniqueness of the library is accompanied by an emphasis on student-centred learning (promoted by the EHEA). As shown in the attached graphic Scheme 3, a compositional comparison can be identified between the FUB Campus and the musical work *Tunnel Spiral*; Stockhausen created a work where the movement of sound would be spiral.

### 3.2. The Social Projection of Fashion and Its Potential Influence as an Inspiration Resource for the Innovative Use of Urban Spaces for Human Education. A Qualitative Methodological approach

Fashion shares compositional qualities with music and architecture. All three present similar learning modalities, born of their creative nature. In this section, some aspects are underlined as valid sources of inspiration for avant-garde ways of using places for education in the three disciplines. Innovation in education induces the activation of physical spaces that behave as original stages. In addition to the common compositional features enunciated, fashion and architecture (like music) have to commit to the social projection of their works. For this reason, the traditional material body of communities (the city) is essential. As expressed earlier, the three disciplines use traditional pedagogical formats (masterly lesson, for instance), but also innovative ones, such as project-based learning or student presentations, among others. In the field of fashion, recent examples of student presentations can be extrapolated to music and architecture. Student presentations are a set of experiences that can be associated with students’ social projection based on innovative public presentations of student academic outcomes. To properly focus the creative keys of these experiences, it must be underlined that fashion, like architecture and music, manifests a close link with nonverbal language in order to express the human commitment to culture. Innovative learning experiences in fashion are fully applicable in music and architecture. It should be noted that these experiences have been the result of adaptation to the place, understood as a composition mechanism. They have been housed in urban areas with a high heritage load, acting as scenarios for educational progress. Fashion teaching has sought to alienate itself from traditional spaces (classrooms, sewing workshops, or conventional laboratories) to go out to meet society and thus generate a whole wealth of social projection. Runways in fact represent quite a singular case. They compose a spatial and temporal reality wherein diverse geometrical compositions are combined (linear, circular, oval, rectangular, centred, or with random circulation). In this sense, the built space is assembled with musical and light components together with the complementary thematic treatment of the collection (water, wind, fire, earth, or rain, as examples). The catwalk is an innovative learning space for fashion and architecture, forging an integral composition. This staging format, which takes place outside the academic centre, merges both disciplines in an explicit way with a high expressive potential. Furthermore, runways are normally accompanied by musical passages, expressively assembling the three creative disciplines. Catwalks could be branded as a metaphysical experience, since they serve as a link between the universe of the couturier’s creativity and the perceptual territory of those who attend the parade. 

#### 3.2.1. Relevant Illustrations (Catwalks): Outcomes of the Qualitative Methodology Approach

After outlining certain theoretical principles that link fashion and architecture, a set of contemporary cases that are of notable interest are studied. It should be emphasized that fashion is an urban phenomenon, both because of its nature and because of its potential to contribute to the renewal of contexts [42,43]. These cases consisted in the celebration of urban catwalks through which students exhibited their designs. Thus, an innovative learning modality took shape (student presentations) but was enriched with a key aspect: the incorporation of high-value architectural pieces as urban settings that hosted them [44]. 

#### 3.2.2. Student Urban Catwalk at the Conde Duque Cultural Centre (Madrid, Spain)

The Centro Superior de Diseño de Moda de Madrid (CSDMM), attached to the Universidad Politécnica de Madrid (UPM), organized a student catwalk in 2017 in quite a singular urban location. As an innovative feature, it took place outside the traditional seats of the CSDMM. The academic runway was performed along the streets and open spaces surrounding a unique piece of heritage: the Conde Duque Cultural Centre. This monumental building, formerly a military structure, is located in the Central District of Madrid. Some relevant complementary keys of innovation should be highlighted. First, the runway was located within a valuable heritage setting, the aforementioned Conde Duque Cultural Centre. Designed in 1717 by Pedro de Ribera, it was renovated in 1981 by Cano Lasso, who majorly transformed it in order to house the new cultural function. Second, the catwalk was designed as an integral event where architecture, music, and Fashion merged, as the staging of the designs was adorned with a modern musical accompaniment that resonated in the external metropolitan areas. Third, the students’ works were projected onto the adjacent streets and urban areas, expressing the vocation of fashion to project its academic outcomes towards the social environment.

#### 3.2.3. Student Urban Catwalk at the Museum of Art (Savannah, GA, USA) 

The Savannah College of Art and Design (SCAD) established synergies through a set of social and cultural facilities distributed along the orthogonal grid of the city; one such facility is the Museum of Art, built in 1853 as a facility serving the Central Georgia Railway. It is a neo-Greek style structure that uses brick as the predominant material and was declared a National Historic Landmark. In 2014, a catwalk of students (graduates of the Bachelor of Fine Arts, Master of Arts, and Master of Fine Arts programs) was held in said cultural piece. As an experience of teaching innovation, it used a heritage environment with a great artistic–cultural load (the Museum of Art). With this, the overlapping of academic and artistic results was achieved in an urban sector where there were other museum facilities (e.g., the Savannah History Museum, Georgia State Museum, and Ralph Gilbert Civil Rights Museum). The students’ walkway was located inside the Museum of Art building, adjusting the exhibition itinerary to the pre-existing spaces, and was carried out along tributaries of an orthogonal grid with a predominance of linearity, adjusted to the adjacent road (Turner Boulevard). As an added sample of synergies with the social and urban context, SCAD promoted the building’s rehabilitation process as a sign of its commitment to the students and the surrounding social community. In this way, the institution promoted the reform of old urban-architectural structures in Savannah [45]. 

#### 3.2.4. Student Urban Catwalk at the Via Tornabuoni (Florence, Italy) 

The Istituto Marangoni has a solid international presence, with seats in Florence, Milano, Paris, London, Mumbai, and Miami. In the former location, it organized in 2019 an expressive student urban runway in a city for which fashion is a sign of identity [46]. As part of its innovative characteristics, the runway was displayed on one of the arteries of the Florentian historic centre, the Via Tornabuoni. The students of the third course of the Fashion Design Programme exhibited their designs along this emblematic street. Currently, this street is where the Istituto Marangoni has its headquarters: the Palazzeto Tornabuoni, an iconic piece of heritage built in the 18th century. A wooden surface was arranged to enhance the linear display of the runway. This configured globally a multiple display, innovative in its configuration: the temporary exhibition sequence of the students’ designs; the superimposed musical melody; and finally, the spatial concatenation of emblematic heritage pieces, which seemed to “parade”, accompanying the models. The facades of several Palazzos (Strozzi, Viviani dell Robbia, and Larderel) constituted an outstanding architectural sequence along the runway. By holding the academic runway in an urban context with such a high aesthetic load, the Istituto Marangoni managed to project the academic works of its students in a singular and intense way in the sociourban context. One quality shared by the cases collected in this section is the incorporation of elements of heritage value to build scenarios of learning innovation. 

## 4. Discussion

### Findings after the Qualitative Methodological Approach and Discussion Thereof

The qualitative methodology was the most suitable for the research process carried out. Its essence corresponded in full coherence with the study of the recognizable compositional and formative synergies among the three areas of knowledge. This methodology is not especially interested in analysing a phenomenon by limiting it, but rather considers all the surrounding elements. In the case of architecture, music, and fashion, the physical space is a critical part of such a series of adjacent elements.

The previous sections of this article discuss an investigation of the compositional and formative synergies among architecture, music, and fashion. The basic outcome of the comparative study was that those synergies generated positive impacts on the design of physical spaces that can house innovative learning activities in higher education. 

In the case of the music–architecture link, the main finding was that melodies could serve for the ideation of innovative planning ideas at the campus scale (although they could be valid as well for other scales, such as buildings or classrooms) and vice versa. Furthermore, they had positive consequences for pedagogy, as the three campuses analysed are distinguished by their high levels of quality and formative innovation. Music can also inspire students’ design skills. Regarding the formative synergies between fashion and architecture, the most relevant finding was that they serve for the ideation of innovative learning modalities on an urban scale, which implies the innovative use of spaces. Analogously to what was pointed out in the three campuses, in the case of student runways, the academic institutions that promoted them are highly prestigious. A simple extrapolation of the aforementioned teaching innovations in fashion (presentation of student shows in urban areas) to the field of architecture can be mentioned. At CEU San Pablo University, exhibitions of student work have held for the public in unique spaces (such as the FabLab, coordinated with MIT and belonging to an international network that includes IIT) in which the intrinsic value of the students’ work was joined by the aesthetic and heritage contribution of the place wherein it was exhibited.

Given that the learning event must be internalized from a comprehensive perspective (pedagogical, human, and spatial relationship), the comparisons made among the three disciplines highlight that the potential benefits derived from the synergies among them assume the human dimension (that is, of the crystallization of “places” and not mere “spaces)” [47,48]. This is key to reinforcing the quality of higher education. Understanding how the human being experiences training actions, and the spaces associated with those actions, is key to increasing global quality and learning innovation. As indicated by the previously discussed case studies, creative resources such as the synergies among architecture, music, and fashion foster innovation.

This text attempts to cover in unison aspects of interaction among architecture, music, and fashion. In addition to the characteristics already extolled, an analogously common aspect must be added: the sensory education provided by all three fields, that is, the formative potential they possess derived from the mere fact of being enjoyed by those who experience them [49]. This is evident in the case of fashion designs, musical works, and architectural pieces that express ideas and values through a system of three-dimensional-geometric signs. Emphasis should be placed on the city as a provider of high-value heritage settings capable of inducing learning innovation. The city can be understood as a complex physical and cultural fact that pervasively influences the daily life of the community that enjoys it. 

Learning innovation is an obvious positive result of the commitment to modernity that characterizes the experiences described. Higher education makes use of urban heritage as a stimulus for creativity, for potential social projection. The physical space is thus involved as a key to innovation in parallel with academic progress itself, since the academic works of students are exposed. Reviewing some recent cases that illustrate this contemporary trend indicates that the essence of this trend is fully extrapolated to the sphere of music and architecture. The public exhibition of student works (architectural projects, music works, or fashion costumes) making use of urban settings endowed with a significant cultural load ultimately constructs a comprehensive training event. In addition to projecting learning results onto the social environment, the urban-architectural scenario itself provides a highly significant framework that increases the motivation of the students. In short, a learning innovation strategy can be generated from a comprehensive approach that reinforces the synergies among fashion, music, and architecture.

## 5. Conclusions

### 5.1. Synergies, Physical Space, and Learning Innovation

Innovation in learning processes entails a deep transformation in higher education. As explained throughout the text, it can be induced by synergies (synergy can be understood as an added value that emerges from the overlapping of diverse areas of knowledge). Such renewed dynamics lead faculty along an itinerary that assigns them a role as a catalyst for creative activities. Some solid conclusions can be expressed. First, music–architecture compositional synergies generate learning innovation in two ways: as a source of inspiration for the design of a campus of academic excellence, and as a creative design resource for future architects. Second, fashion–architecture formative synergies generate learning innovation through the use of the city’s architectural heritage as a setting for creative pedagogical modalities (exhibition of students’ work, to which the aesthetic value of said urban heritage is added).

Given this, it is essential to assess the metamorphosis that physical space must undergo. Human beings live through their multiple and varied interactions with the environment. Thus, for learning innovation, the physical space must play a key role. One of the most solid conclusions of this article is that, as a result of the synergies among architecture, music, and fashion, outstanding levels of formative quality have been reached. That is clear both in the configuration of the campus and in the creative use of areas belonging to the urban heritage. It was confirmed that educational quality is closely linked to the quality of physical spaces. This confirmation emanated from comparative readings of the three campuses (IIT, Espoo, and FUB) and the three urban environments (Madrid, Savannah, and Florence) studied.

At this moment of paradigm shift in terms of learning formats, the city is called to be incorporated as one more classroom area, endowed with an enormous cultural power. The irruption of educational activities in the public space increases the visibility of the university in everyday life. Cities can play a relevant “educational” role by contributing their invaluable social and heritage value. The socio-urban environment offers enormous opportunities to promote learning innovation in higher education. The transfer in compositional matter among the three disciplines studied is similarly a powerful source of inspiration for each of them, as was evident in the cases selected.

### 5.2. From Qualitative Methodology to Proactive Comprehensive Outcomes

One conclusion of a more proactive profile that can be drawn from the previous discussion is that progress in innovation (pedagogical or spatial) can arise from the synergies among various disciplines (such as architecture, music, and fashion). This conclusion is connected to the methodological approach (qualitative) in these creative disciplines. One reason for choosing this qualitative methodology was that theoretical or subjective aspects can be not only a source of knowledge but a sort of object of science in themselves. Furthermore, the qualitative methodology made it possible to incorporate findings that were not initially foreseen. This helped to better understand the phenomenon studied, as well as creating a source for potential applications in different disciplines.

The teaching innovation guidelines that may arise from the synergies among the three disciplines (architecture, music, and fashion) have an added potential, which is to induce entrepreneurship in future graduates; this matter was recently dealt with [50]. As a suggestion to inspire future lines of research, qualitative methodology and case studies may also provide benefits in terms of theoretical results that can be extrapolated to the professional sphere and in terms of connections among the three disciplines analysed [51]. For such purposes, it would be advisable to use the case study methodology [52] because it was effective in preparing the research on which this text was based. The synergies among architecture, music, and fashion have consisted of transfers of various content and attitudes among these disciplines, which has provided global enrichment for all of them in terms of creativity. This fact corresponds coherently with the use of a qualitative methodology. Qualitative methodology was quite useful as a complementary method of generating theories to open up future lines of research.

At this point, it is appropriate that, by way of conclusions, a specific emphasis is made on this concept: Professor Csíkszentmihály noted that creative people feel happy because, among other personal attitudes and feelings, they come to transcend the limits of a domain, entering in a diverse universe of knowledge [53]. This attitude of going beyond the limits of an area of knowledge to access a scenario of synergies has undoubtedly characterized the set of experiences described in the present text. For all these reasons, and with an eye to the future, this type of educational–spatial innovation dynamic can induce different synergies. Creative synergies among architecture, music, and fashion were revealed in this paper. However, the procedures herein can be extrapolated to other areas of knowledge, thus inducing a dynamic of global progress that benefits various institutions of higher education. To open that new research field, a preliminary approach to the specificities of new synergies would be necessary.

## Data Availability

Not Applicable.
